# Association between phthalate exposure and lower handgrip strength in an elderly population: a repeated-measures study

**DOI:** 10.1186/s12940-016-0176-2

**Published:** 2016-08-31

**Authors:** Kyoung-Nam Kim, Mee-Ri Lee, Yoon-Hyeong Choi, Hyojung Hwang, Se-Young Oh, ChoongHee Park, Yun-Chul Hong

**Affiliations:** 1 Department of Preventive Medicine, Seoul National University College of Medicine, 28 Yongon-Dong, Chongno-Gu, Seoul, Republic of Korea; 2 Public Health Medical Service, Seoul National University Hospital, Seoul, Republic of Korea; 3 Department of Preventive Medicine, Gachon University Graduate School of Medicine, Incheon, Republic of Korea; 4 Department of Food and Nutrition, Research Center for Human Ecology, College of Human Ecology, Kyung Hee University, Seoul, Republic of Korea; 5 Environmental Health Research Division, Environmental Health Research Department, National Institute of Environmental Research, Incheon, Republic of Korea; 6 Institute of Environmental Medicine, Seoul National University Medical Research Center, Seoul, Republic of Korea; 7 Environmental Health Center, Seoul National University College of Medicine, Seoul, Republic of Korea

**Keywords:** Elderly, Handgrip strength, Omega-6 to omega-3 ratio, Phthalate

## Abstract

**Background:**

Decreased muscle strength can lead to adverse health outcomes in the elderly. A potential association between phthalate exposure and muscle strength was suggested previously, but has not been investigated directly. We hypothesized that phthalate exposure is associated with lower handgrip strength and that the association is modified by the dietary omega-6 to omega-3 ratio.

**Methods:**

We analyzed 1,228 participants (≥60 years of age) recruited in Seoul and Asan, Republic of Korea. The study participants were surveyed up to three times between 2012 and 2015. At every survey, we collected urine samples and measured handgrip strength twice for each hand. The associations between urine phthalate metabolite concentrations and handgrip strength were evaluated using linear mixed models. Based on dietary information from 391 individuals who participated in the first survey in Seoul, we evaluated the heterogeneity of the association for those with high and low omega-6 to omega-3 ratios, using 8.81 (the 75th quantile) as a cutoff value.

**Results:**

Log-transformed creatinine-adjusted concentrations of mono-(2-ethyl-5-oxohexyl phthalate (MEOHP), mono-(2-ethyl-5-hydroxyhexyl) phthalate (MEHHP), and mono-n-butyl phthalate (MnBP) were inversely associated with all measured handgrip strengths (β = −0.69 to −0.42, all *p*-values < 0.05). Associations between phthalate biomarkers and handgrip strength did not differ by sex. When the dietary subgroup was stratified by the omega-6 to omega-3 ratio, the associations were stronger among participants with high ratios.

**Conclusions:**

We found inverse associations between phthalate biomarkers and handgrip strength in the elderly; this association was modified by the dietary omega-6 to omega-3 ratio.

**Electronic supplementary material:**

The online version of this article (doi:10.1186/s12940-016-0176-2) contains supplementary material, which is available to authorized users.

## Background

Phthalates are high-volume production industrial chemicals that are used to increase the plasticity and softness of plastics and to maintain scents in a wide range of personal care products. Phthalates can easily be released into the environment from plastics and absorbed by humans through ingestion, inhalation, or dermal contact [1]. The use of phthalates is ubiquitous, and a detectable level of urinary phthalate metabolites is reportedly present in more than 75 % of the U.S. population [2]. Previous studies also reported that urinary metabolites of di-(2-ethylhexyl) phthalate (DEHP), a widely-used phthalate, are detectable in more than 75 % of population-based samples collected from the Republic of Korea and other Asian countries [3, 4].

The levels of phthalate exposure that are observed in developed countries have been associated with increased oxidative stress and inflammation [5-7]. Oxidative stress and inflammation can induce broad spectrums of pathologic conditions in many organs and tissues, and are thought to mediate many adverse health outcomes associated with phthalate exposure [8]. The muscle is one of the tissues that can be affected, and is reportedly damaged directly by reactive oxygen species [9] and persistent proinflammatory states [10], leading to decreased muscle strength [11]; this is especially true in the elderly because of age-related functional decline and increased oxidative stress [12].

Decreased muscle strength may reduce physical activity levels [13] and increase falls and fractures [14], which in turn induce further decreases in muscle strength. Handgrip strength is a reliable surrogate for whole-body muscle strength [15], including of the lower extremities [16], and previous studies have reported that decreases in handgrip strength are associated with increased mortality [17], mobility declines [18], functional disability [19], and cognitive deterioration [20] in the elderly.

Omega-3 and omega-6 polyunsaturated fatty acids are essential nutrients and should be ingested as part of the normal diet. Because the metabolic pathways for omega-3 and omega-6 fatty acids share the same enzymes, and because the relative amounts of these fatty acids may determine the availability of associated enzymes, the balance between omega-3 and omega-6 fatty acids in the diet is important. A high omega-6 to omega-3 ratio is reportedly related to inflammation [21] and to augmentation of inflammatory responses induced by environmental pollutants [22], suggesting that the ratio of these fatty acids might modify any association between phthalate exposure and handgrip strength.

We hypothesized that concentrations of urinary phthalate metabolites would be inversely associated with handgrip strength among the elderly. We also hypothesized that the association would be stronger among individuals with a high dietary omega-6 to omega-3 ratio than among those with a low ratio. We assessed these hypotheses in an elderly population using measures of urinary phthalate metabolite levels, handgrip strength, and dietary records.

## Methods

### Study design and population

The Korean Elderly Environmental Panel II (KEEP II) study is a repeated-measures study conducted between 2012 and 2015 to investigate the associations between environmental risk factors and health outcomes in the elderly. We recruited 1,253 noninstitutionalized elderly citizens who regularly visited community welfare centers in Seoul and Asan, Republic of Korea, and volunteered to participate in the study. The inclusion criteria were an age of 60 years or older and the ability to communicate with interviewers. During the study period, three surveys were performed at approximately 1-year intervals. When those who participated in the previous survey(s) were not followed up, new participants were recruited to meet the survey objective of 400 participants each in Seoul and Asan. Therefore, of the recruited subjects, 435 (34.7 %) participated in the survey only once (either the first, second, or third survey), 491 (39.2 %) participated twice, and 327 (26.1 %) participated three times. There were no seasonal differences between the surveys with respect to sites and repeated measurements. At each survey, trained interviewers obtained information about demographic characteristics, socioeconomic status, medical and family history, and lifestyle factors using structured questionnaires. Blood samples, urine samples, handgrip strength measurements, and anthropometric measurements were also collected at every survey. Using the collected biospecimens, levels of mono-(2-ethyl-5-oxohexyl) phthalate (MEOHP), mono-(2-ethyl-5-hydroxyhexyl) phthalate (MEHHP), mono-n-butyl phthalate (MnBP), blood lead, mercury, cadmium, and urinary 3-phenoxybenzoic acid were analyzed. All participants submitted written statements of informed consent, and the Institutional Review Board of Seoul National University Hospital approved the study protocol (C-1209-006-424). This study was performed in accordance with the Declaration of Helsinki.

Among the 1,253 recruited individuals, we excluded those for whom there was no information regarding urinary phthalate metabolite concentration (*n* = 6), handgrip strength (*n* = 5), or body mass index (BMI; *n* = 14); data for the remaining 1,228 participants were analyzed in regression models to evaluate associations between urinary phthalate metabolites and handgrip strength. Although dietary information was obtained only at the first survey in Seoul (*n* = 400), we treated this information as being time-independent for our analyses of the second and third surveys because the study period was relatively short (<2.5 years). Our analysis of the interaction between urinary phthalate metabolites and the omega-6 to omega-3 ratio was restricted to the subset of study participants. Data of 391 participants in the Seoul survey for whom the required dietary information was available were used in this analysis.

### Urinary phthalate metabolites

We collected urine samples between 10:00 AM and 12:00 PM and stored them at −20 °C until analysis. To reduce the possibility of contamination, monoester phthalate metabolites, such as MEOHP, MEHHP, and MnBP, were measured instead of their parent compounds. Urinary phthalate metabolites were analyzed using ultra-high performance liquid chromatography tandem mass spectrometry (Nexera X2; Shimadzu, Kyoto, Japan) according to a previously reported procedure [6]. The limits of detection (LODs) for MEOHP, MEHHP, and MnBP were 0.32 μg/L, 0.20 μg/L, and 0.35 μg/L, respectively. We substituted urinary phthalate metabolite levels below the LOD with the LOD divided by the square root of 2 [23]. Because MEOHP and MEHHP are metabolized from the same parent compound, DEHP, a summed measure (∑DEHP) was calculated by adding the molar sums of MEOHP and MEHHP. We adjusted the urinary phthalate metabolite concentrations by dividing them by the creatinine concentration from the same urine sample to consider the different urinary excretion rates of study participants. The urinary creatinine level was determined using the kinetic Jaffe method (Cobas 8000 C702; Roche Diagnostics, Mannheim, Germany).

### Handgrip strength

We measured handgrip strength using a regular-sized grip dynamometer (Hand Grip Meter 6103, Tanita, Tokyo, Japan). After the examiner showed the participants how to properly use the dynamometer, measurements were obtained while each participant kept his or her shoulder adducted and elbow flexed at 90°. Study participants conducted two attempts per hand, with a 1-min rest between each attempt to reduce the effect of repetition fatigue [24].

### Dietary intake

We assessed the dietary intake of the study participants using a semi-quantitative food frequency questionnaire that assesses the frequency of consumption (categorized into nine categories from “rarely eaten” to “more than three times per day”) and portion size (categorized as small, average, or large) of 118 food items during the previous year. The amounts for each item were transformed into grams, and daily nutrient intake was estimated using the Computer Aided Nutritional Analysis Program 4.0 for professionals (CAN-pro 4.0, Korean Society of Nutrition, Seoul, Korea).

### Covariates

From the structured questionnaire used at each survey, we obtained information regarding the participant’s age (years), sex, tobacco smoking (nonsmoker, ex-smoker, or smoker), alcohol drinking (no drinking, ex-drinker, or drinker), moderate physical activity (no exercise, ≤3 times/week, or ≥4 times/week), monthly income (<US$450, US$450–1,349.9, or ≥ US$1,350), education level (<elementary school, elementary or middle school, or ≥ high school), city of residence (Seoul or Asan), and comorbidity status (the number of the following self-reported chronic diseases, categorized as 0, 1, 2, or ≥3: hypertension, diabetes mellitus, chronic obstructive pulmonary disease, osteoarthritis, stroke, coronary heart disease, and cancer). We calculated BMI (kg/m^2^) from the participant’s height and weight. Categorical covariates with missing values had a missing indicator category.

We also measured blood lead, mercury, cadmium, and urinary 3-phenoxybenzoic acid levels at every survey and included them as covariates in the sensitivity analysis. Blood lead and cadmium levels were analyzed using graphite-furnace atomic absorption spectrometry with Zeeman background correction (AAnalyst 800; Perkin Elmer, Shelton, CT, USA). Blood mercury level was determined using the gold-amalgam collection method with a direct mercury analyzer (DMA-80; Milestone, Bergamo, Italy). Urinary 3-phenoxybenzoic acid level was analyzed using gas chromatography-mass spectrometry (Clarus 680 T; Perkin Elmer, Shelton, CT, USA).

### Statistical analysis

Because creatinine-adjusted phthalate metabolite concentrations (μg/g creatinine) and ∑DEHP followed log-normal distributions, we log-transformed these variables for further analyses. As the main outcome, we used the average value of the right and left handgrip strengths because of the high correlations between measurements (see Additional file 1: Table S1).

We assessed the associations between each urinary phthalate metabolite and handgrip strength using linear mixed models with a first-order autoregressive variance-covariance matrix after visually examining the shapes of the associations using nonparametric analyses with generalized additive mixed models. We also conducted sex-stratified analyses because previous studies suggested sex-specific effects of phthalates due to endocrine disruption [25, 26].

We stratified the participants with dietary intake information (*n* = 391) into those with a dietary omega-6 to omega-3 ratio ≥8.81 (the 75th quantile value) and those with a ratio <8.81. The associations between phthalate biomarkers and handgrip strength were also evaluated in each stratum using the linear mixed models.

All models were adjusted for potential confounders that had been selected a priori, based on biological considerations and previous literature reviews; confounders included age, sex, tobacco smoking, alcohol drinking, moderate physical activity, monthly income, education level, city of residence, and comorbidity status. Excepting sex and city of residence, the covariates were included in the analyses as time-varying variables.

In sensitivity analyses, we constructed multiple pollutant models that included lead, mercury, cadmium, 3-phenoxybenzoic acid, ∑DEHP, MnBP, and the same covariates in the main model to control for potential confounding by other environmental exposures. We utilized the least absolute shrinkage and selection operator (LASSO) regression methods due to their robustness with regard to regression coefficient estimation and ability to identify coefficients associated with variables of interest [27]. In addition, we supplemented our main analysis of the 75th quantile of the omega-6 to omega-3 ratio (8.81) with analyses stratified by the median (7.54) and 90^th^ quantile (9.94) values because the optimal ratio has not been determined and may vary according to outcome [28, 29]. Because the present results could be affected by conditions such as stroke, osteoporosis, or osteoarthritis, we evaluated the robustness of the results after excluding those who had been diagnosed with stroke (*n* = 47), osteoporosis (*n* = 157), or osteoarthritis (*n* = 297). Finally, to address potential biases due to selective loss to follow-up, we weighted the follow-up observations by the inverse predicted probability of follow-up [30], and repeated the analyses.

All analyses were performed with SAS version 9.4 (SAS Institute Inc., Cary, NC, USA) and R version 3.2.0 (The Comprehensive R Archive Network: http://cran.r-project.org).

## Results

Table 1 shows the baseline characteristics and urinary phthalate metabolite levels among the 1,228 included individuals. The average age was 75.0 years, and the majority of the study participants were women (69.5 %), nonsmokers (81.9 %), non-drinkers (72.2 %), and those who did not exercise regularly (62.9 %). The concentrations of MEOHP and MEHHP were elevated among those who were older, smokers, did not exercise regularly, had lower income and education levels, and had more chronic diseases, while the concentration of MnBP showed no clear relationships with these features.

**Table 1:** Concentrations of Phthalate Metabolites (μg/L) and Handgrip Strength (kg) According to baseline characteristics of the study population in the korean elderly environmental Panel II Study (2012–2015)

Baseline covariates	Total^a^ (*n* = 1,228)	1st survey (*n* = 758)	2nd survey (*n* = 767)	3rd survey (*n* = 782)	Handgrip strength^b^ (AM ± SD)	MEOHP (GM ± GSD)	MEHHP (GM ± GSD)	MnBP (GM ± GSD)
Age (years)	75.0 ± 6.1	74.7 ± 5.9	75.7 ± 5.8	76.0 ± 5.8				
60–69	244 (19.9)	153 (20.2)	116 (15.1)	111 (14.2)	22.0 ± 6.9	13.5 ± 2.8	18.8 ± 2.8	27.9 ± 2.1
70–79	694 (56.5)	444 (58.6)	459 (59.8)	453 (57.9)	20.4 ± 7.5	15.4 ± 2.4	20.7 ± 2.5	28.2 ± 2.2
≥80	290 (23.6)	161 (21.2)	192 (25.0)	218 (27.9)	17.0 ± 7.0	17.6 ± 2.4	24.3 ± 2.4	28.5 ± 2.2
Sex								
Men	374 (30.5)	237 (31.3)	197 (25.7)	216 (27.6)	27.7 ± 6.5	16.5 ± 2.3	22.6 ± 2.4	29.8 ± 2.1
Women	854 (69.5)	521 (68.7)	570 (74.3)	566 (72.4)	16.5 ± 4.9	15.0 ± 2.6	20.5 ± 2.6	27.6 ± 2.2
Tobacco smoking								
Nonsmoker	1,007 (82.0)	615 (81.1)	650 (84.8)	645 (82.5)	18.3 ± 6.6	15.3 ± 2.5	20.8 ± 2.5	27.8 ± 2.2
Ex-smoker	161 (13.1)	108 (14.3)	87 (11.3)	111 (14.2)	27.5 ± 7.0	16.0 ± 2.3	22.0 ± 2.6	31.9 ± 2.2
Smoker	60 (4.9)	35 (4.6)	30 (3.9)	26 (3.3)	26.4 ± 6.9	18.1 ± 2.1	24.0 ± 2.3	26.5 ± 2.3
Alcohol drinking								
No drinking	886 (72.2)	538 (71.0)	579 (75.5)	427 (54.6)	18.2 ± 6.5	15.6 ± 2.5	21.2 ± 2.5	27.5 ± 2.2
Ex-drinker	95 (7.7)	60 (7.9)	59 (7.7)	109 (13.9)	23.8 ± 7.2	15.4 ± 2.2	20.6 ± 2.5	33.0 ± 2.3
Drinker	243 (19.8)	159 (21.0)	122 (15.9)	246 (31.5)	24.4 ± 8.5	15.4 ± 2.4	21.1 ± 2.6	29.4 ± 2.0
Did not answer	4 (0.3)	1 (0.1)	7 (0.9)	0 (0)	17.7 ± 1.6	9.6 ± 10.5	22.7 ± 4.8	21.6 ± 3.9
Moderate physical activity								
No exercise	772 (62.9)	486 (64.1)	430 (56.1)	403 (51.5)	19.2 ± 7.9	16.0 ± 2.4	22.0 ± 2.5	26.5 ± 2.2
≤3 times/week	206 (16.8)	105 (13.9)	190 (24.8)	190 (24.3)	19.3 ± 6.3	15.1 ± 2.4	19.8 ± 2.5	31.7 ± 2.0
≥4 times/week	246 (20.0)	163 (21.5)	147 (19.2)	189 (24.2)	22.4 ± 6.7	14.3 ± 3.0	19.6 ± 2.7	30.8 ± 2.2
Did not answer	4 (0.3)	4 (0.5)	0 (0)	0 (0)	20.2 ± 3.9	22.7 ± 1.7	30.1 ± 1.5	44.8 ± 1.4
Monthly income (US $)								
<450	589 (48.0)	316 (41.7)	426 (55.5)	327 (41.8)	19.2 ± 7.4	16.8 ± 2.3	23.0 ± 2.4	27.7 ± 2.2
450–1,349	173 (14.1)	75 (9.9)	120 (15.7)	264 (33.8)	21.9 ± 8.3	15.7 ± 2.7	22.7 ± 2.5	30.5 ± 2.2
≥1,350	126 (10.3)	85 (11.2)	92 (12.0)	87 (11.1)	23.4 ± 7.5	12.4 ± 2.7	17.2 ± 2.7	27.1 ± 2.2
Did not answer	340 (27.7)	282 (37.2)	129 (16.8)	104 (13.3)	18.8 ± 6.6	14.4 ± 2.6	18.9 ± 2.7	28.4 ± 2.1
Education level								
Less than elementary school	399 (32.5)	224 (29.6)	254 (33.1)	272 (34.8)	16.1 ± 6.1	16.8 ± 2.3	22.8 ± 2.3	27.6 ± 2.2
Elementary or middle school	598 (48.7)	371 (48.9)	374 (48.8)	373 (47.7)	20.4 ± 7.0	15.5 ± 2.6	21.4 ± 2.6	28.2 ± 2.2
High school or higher	231 (18.8)	163 (21.5)	139 (18.1)	137 (17.5)	24.9 ± 7.7	13.4 ± 2.6	17.8 ± 2.6	29.5 ± 2.1
City of residence								
Seoul	554 (45.1)	391 (51.6)	392 (51.1)	396 (50.6)	19.9 ± 6.6	12.2 ± 2.5	15.9 ± 2.5	30.2 ± 2.1
Asan	674 (54.9)	367 (48.4)	375 (48.9)	386 (49.4)	19.9 ± 8.1	18.8 ± 2.3	26.6 ± 2.4	26.7 ± 2.2
Body mass index (kg/m^2^)	23.7 ± 3.2	24.0 ± 2.9	23.8 ± 3.3	24.2 ± 3.3				
<23	513 (41.8)	292 (38.5)	311 (40.6)	295 (37.7)	18.9 ± 7.5	15.8 ± 2.5	21.0 ± 2.6	26.5 ± 2.2
23–24	319 (26.0)	207 (27.3)	189 (24.6)	193 (24.7)	21.5 ± 7.5	14.8 ± 2.6	20.4 ± 2.6	28.2 ± 2.1
≥25	396 (32.3)	259 (34.2)	267 (34.8)	294 (37.6)	19.8 ± 7.3	15.7 ± 2.4	21.9 ± 2.3	30.6 ± 2.2
Comorbidity status^c^								
0	351 (28.6)	208 (27.4)	189 (24.6)	146 (18.7)	20.5 ± 7.6	14.1 ± 2.7	18.9 ± 2.7	27.2 ± 2.1
1	499 (40.6)	329 (43.4)	282 (36.8)	280 (35.8)	20.3 ± 7.9	16.0 ± 2.4	21.8 ± 2.4	28.5 ± 2.1
2	291 (23.7)	183 (24.1)	215 (28.0)	213 (27.2)	19.2 ± 6.9	15.7 ± 2.6	21.8 ± 2.6	29.3 ± 2.3
≥3	87 (7.1)	38 (5.0)	81 (10.6)	143 (18.3)	17.7 ± 6.4	17.9 ± 2.3	24.6 ± 2.3	27.5 ± 2.2

*AM* arithmetic mean, *SD* standard deviation, *GM* geometric mean, *GSD* geometric standard deviation, *MEOHP* mono-(2-ethyl-5-oxohexyl) phthalate, *MEHHP* mono-(2-ethyl-5-hydroxyhexyl) phthalate, *MnBP* mono-n-butyl-phthalate

^a^Baseline characteristics of the participants included in analyses are presented. Values are presented as *n* (%) or means ± standard deviations. ^b^Average of the right handgrip strengths measured at the first and second attempts. ^c^The number of chronic diseases that were self-reported as being present: hypertension, diabetes mellitus, chronic obstructive pulmonary disease, osteoarthritis, stroke, coronary heart disease, stomach cancer, colon cancer, liver cancer, breast cancer, uterine cancer, or other cancer

Geometric means of MEOHP, MEHHP, and MnBP at enrollment were 15.48 μg/L, 21.11 μg/L, and 28.23 μg/L, respectively (see Additional file 1: Table S2), which are comparable to previously reported levels in the Republic of Korea [6, 7], but higher than those in the United States [26]. The levels of each phthalate metabolite were correlated at multiple time points (see Additional file 1: Table S3). In addition, correlations among the levels of MEOHP, MEHHP, and MnBP were observed at every survey (*r* = 0.94, *p*-value < 0.01 at the first survey, *r* = 0.90, *p*-value < 0.01 at the second survey, and *r* = 0.92, *p*-value < 0.01 at the third survey for MEOHP and MEHHP, which were the most strongly correlated; *r* = 0.44, *p*-value < 0.01 at the first survey, *r* = 0.26, *p*-value < 0.01 at the second survey, and *r* = 0.36, *p*-value < 0.01 at the third survey for MEHHP and MnBP, which were the most weakly correlated) (see Additional file 1: Table S4).

**Fig. 1:**
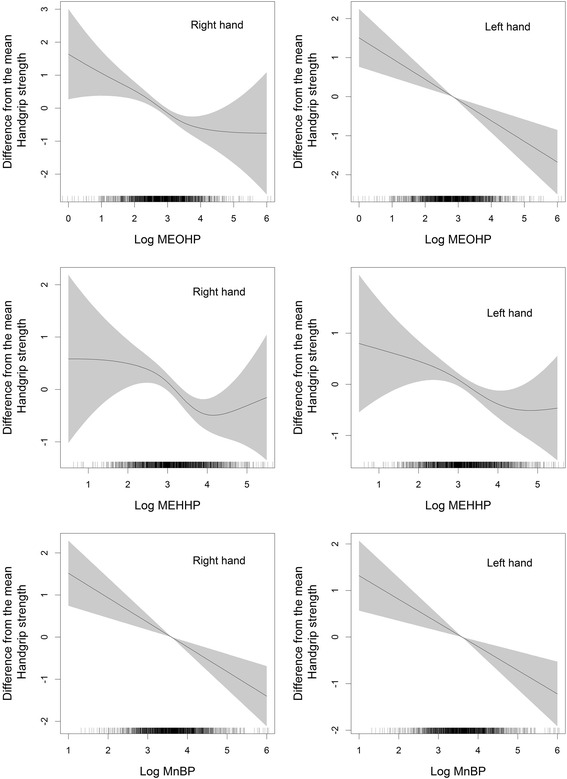
Penalized regression spline of log-transformed creatinine-adjusted urinary phthalate metabolite concentrations and handgrip strengths. Solid lines, spline curves; shaded areas, 95 % confidence intervals; MEOHP, mono-(2-ethyl-5-oxohexyl) phthalate; MEHHP, mono-(2-ethyl-5-hydroxyhexyl) phthalate; MnBP, mono-n-butyl-phthalate. The models were adjusted for age, sex, smoking status, alcohol consumption, physical activity, monthly income, education level, city of residence, body mass index, and comorbidity status

We found an inverse linear association between urinary phthalate biomarkers and handgrip strengths in the penalized regression spline analyses (Fig. 1). Log-transformed creatinine-adjusted concentrations of MEOHP, MEHHP, and MnBP were inversely associated with right and left handgrip strengths after adjusting for potential confounders (Table 2). The results were similar for ∑DEHP (β = –0.69, 95 % confidence interval: –0.95, –0.35 for right hand; β = –0.55, 95 % confidence interval: –0.85, –0.25 for left hand). In multiple pollutant models, consistent associations between phthalate biomarkers and handgrip strengths were observed (see Additional file 1: Table S5). The associations between phthalate metabolite levels and handgrip strength were not different between men and women (all *p*-values for interaction > 0.10; Table 2).

**Table 2:** Associations^a^ between urinary phthalate metabolite concentrations (Log-transformed, μg/g Creatinine) and handgrip strength^b^ (*n* = 1,228) in the Korean elderly environmental panel II study (2012–2015)

	MEOHP		MEHHP		MnBP	
	β	95 % CI	β	95 % CI	β	95 % CI
Total						
Right hand	−0.65	−0.93, −0.36	−0.57	−0.87, −0.28	−0.50	−0.84, −0.17
Left hand	−0.58	−0.86, −0.29	−0.46	−0.76, −0.17	−0.54	−0.87, −0.20
Men						
Right hand	−0.77	−1.44, −0.10	−0.63	−1.29, 0.03	−0.09	−0.91, 0.73
Left hand	−0.85	−1.51, −0.18	−0.65	−1.30, 0.003	−0.45	−1.26, 0.36
Women						
Right hand	−0.60	−0.90, −0.30	−0.54	−0.86, −0.22	−0.65	−0.99, −0.30
Left hand	−0.43	−0.72, −0.13	−0.33	−0.64, –0.01	−0.57	−0.91, −0.23

*MEOHP* mono-(2-ethyl-5-oxohexyl) phthalate, *MEHHP* mono-(2-ethyl-5-hydroxyhexyl) phthalate, *MnBP* mono-n-butyl-phthalate, *CI* confidence interval

^a^Adjusted for age, sex, smoking status, alcohol consumption, physical activity, monthly income, education level, city of residence, body mass index, and comorbidity status. ^b^Average of the handgrip strengths measured at the first and second attempts

The omega-6 to omega-3 ratios in the present study population (median: 7.54; range: 2.38–17.55) were lower than the estimated ratios of 10–20 in typical Western diets [31]. When we stratified the study population into individuals with a ratio ≥8.81 (the 75^th^ quantile value) and those with a ratio <8.81, the association was stronger among those with a high ratio than among those with a low ratio (Table 3). When we changed the cutoff value to 9.94 (the 90^th^ quantile value), the results did not change appreciably; however, when the cutoff value was changed to 7.54 (the median value), the heterogeneity of the associations was attenuated (see Additional file 1: Tables S6 and S7). When we excluded participants who had been diagnosed with stroke, osteoporosis, or osteoarthritis, an appreciable change was not observed (see Additional file 1: Table S8). After weighting the follow-up observations by the inverse probability to attain follow-up, the present results were robust (see Additional file 1: Tables S9 and S10).

**Table 3:** Associations^a^ between Urinary Phthalate Metabolite Concentrations (Log-transformed, μg/g Creatinine) and Handgrip Strength, Stratified by the 75th Quantile of the Omega-6 to Omega-3 Ratio (*n* = 391) in the Korean Elderly Environmental Panel II Study (2012–2015)

		MEOHP			MEHHP			MnBP		
		β	95 % CI	*p*-int	β	95 % CI	*p*-int	β	95 % CI	*p*-int
		Right hand								
Omega-6 to omega-3 ratios^b^	High	−1.78	−2.64, −0.92	0.0265	−1.58	−2.55, −0.60	0.0821	−1.41	−2.35, −0.47	0.2234
	Low	−0.65	−1.11, −0.19		−0.68	−1.18, −0.19		−0.71	−1.29, −0.14	
		Left hand								
Omega-6 to omega-3 ratios^b^	High	−1.28	−2.15, −0.41	0.1819	−1.18	−2.16, −0.21	0.2731	−1.35	−2.29, −0.41	0.1483
	Low	−0.57	−1.02, −0.12		−0.56	−1.05, −0.07		−0.58	−1.14, −0.01	

*MEOHP* mono-(2-ethyl-5-oxohexyl) phthalate, *MEHHP* mono-(2-ethyl-5-hydroxyhexyl) phthalate, *MnBP* mono-n-butyl-phthalate, *CI* confidence interval, *p*-int *p*-value for interaction

^a^Adjusted for age, sex, smoking status, alcohol consumption, physical activity, monthly income, education level, city of residence, body mass index, and comorbidity status. ^b^Omega-6 to omega-3 ratios above and below the 75th quantile value (8.81) are defined as high and low, respectively

## Discussion

We found an inverse association between urinary phthalate metabolite concentrations and handgrip strength in the present study of a noninstitutionalized elderly population. When we stratified the study population according to the dietary omega-6 to omega-3 ratio, which is related to a higher level of inflammation, the association was stronger among those with a high ratio than among those with a low ratio.

In previous studies, phthalate metabolite concentrations comparable to the levels of the current study have been associated with increased oxidative stress and inflammation [5-7]. These associations may be attributable, at least partly, to the affinity of phthalate for peroxisome proliferator-activated receptor-γ (PPAR-γ) [32], which is a nuclear transcription factor that plays an important role in reducing production of pro-inflammatory cytokines. Phthalates have been reported to suppress the activity of PPAR-γ, resulting in a high inflammatory status [32].

Oxidative stress could induce mitochondrial and nuclear DNA damage in muscle cells, resulting in decreased muscle function [9]. Among non-critically ill patients, inflammatory status assessed by the serum level of CRP was inversely associated with handgrip strength [11]. In a prospective longitudinal study of an elderly population, inflammatory markers such as interleukin-6 and CRP were associated with a decrease in handgrip strength during the 3 years of follow-up [10]. Oxidative stress and inflammation are thought to be closely related to the metabolism of muscle protein by suppressing anabolism as a consequence of increased energy demand and consumption [33, 34]. We previously reported an association between phthalate biomarkers and insulin resistance, which was possibly mediated by oxidative stress [6, 7]. Because insulin decreases the breakdown of muscle protein and enhances muscle anabolism [35], the present results could also be attributable to the inhibition of insulin action. In sex-stratified analyses, we observed no sex differences in the associations between phthalates and handgrip strength, which provides further support for the idea that the inverse associations are mainly explained by oxidative stress and inflammation pathways, rather than by an endocrine-disrupting mechanism [25, 26], at least among elderly men and postmenopausal women.

The half-lives of the phthalate metabolites are typically reported as 12–48 h [36], as assessed based on pharmacokinetic studies. Nonetheless, phthalate levels have been reported to be stable over 3–6 months, possibly because of the habitual and persistent exposure to consumer products containing phthalates [37]. Phthalates that have accumulated in fat tissue could be released to other parts of the body, contributing to the stability of exposure, and effectively increasing the half-lives beyond the previously reported levels [17, 38]. Therefore, the current results could be interpreted as indicating that higher phthalate exposures over the course of months were inversely associated with handgrip strength in an elderly population.

The observed associations between phthalates and handgrip strengths may be confounded by the use of products containing phthalates that could also be related to handgrip strength, such as medical devices and pharmaceutical coatings. Medical devices, including intravenous tubing, formula bags, and respiratory masks, may contain di-(2-ethylhexyl) phthalate, which is a parent compound of MEOHP and MEHHP. Pharmaceutical coatings may include dibutyl phthalate, which is a parent compound of MnBP [39]. To reduce the possibility of this confounding, we adjusted the models for comorbidity status, BMI, and physical activity. However, residual or unmeasured confounding remains possible, especially with regard to pathways of exposure. Nonetheless, the analyses that were further controlled for multiple environmental pollutants showed robust associations (see Additional file 1: Table S5), which raises the likelihood of a true association.

The relative amounts of omega-3 and omega-6 fatty acids in the diet could affect the endogenous synthesis of long-chain omega-3 fatty acids (such as eicosapentaenoic acid and docosahexaenoic acid) and omega-6 fatty acids (such as arachidonic acid) because both pathways compete for the same enzymes [40]. Because omega-3 fatty acids are anti-inflammatory, while omega-6 fatty acids are proinflammatory [41], a high omega-6 to omega-3 ratio might enhance the inflammatory response to environmental pollutants and modulate the associations between environmental risk factors and adverse health outcomes. A previous study demonstrated that the associations between prenatal methylmercury exposure and neurodevelopmental outcomes assessed at 20 monthnths of age are only observed among those with a high maternal omega-6 to omega-3 ratio [22]. In line with these studies, we found a modification of the association between phthalate metabolites and handgrip strength at an omega-6 to omega-3 ratio of 8.81 (the 75th quantile value). Although there is no currently recommended ratio between omega-3 and omega-6 fatty acids, the present results suggest that handgrip strength would be especially vulnerable to phthalate exposure for elderly individuals with an omega-6 to omega-3 ratio ≥8.81.

There are some limitations to be considered in this study. First, we conducted the present study on elderly individuals who regularly visited community welfare centers, which are public facilities for elderly citizens in the Republic of Korea, and volunteered to participate. Study participants resided in a community and were not institutionalized because of serious illnesses, disabilities, or reduced mobility. The majority of the study participants were women, nonsmokers, non-drinkers, and relatively healthy, which lowers the external generalizability of the current results, especially to populations with lower physical function and more diseases. Considering previous studies that have reported associations between inflammation and decreased muscle strength among severely ill patients, such as those with sepsis or multiple organ failure [42, 43], possibility that phthalate exposure might lead to increased mortality due to weakened diaphragmatic muscle among individuals with lower physical function and more diseases [44] should be investigated in future studies. Second, although handgrip strength is a reliable indicator for whole-body muscle strength, direct measurement in the lower extremities would be more relevant to assess mobility and physical function in the elderly population [45]. However, handgrip strength has been associated with mobility [18, 46] and functional status [47] in the elderly. In addition, although the potential influence of motivation during measurement has been noted as a weakness of using handgrip strength as a measure [15], we evaluated handgrip strength twice per hand at every survey and found consistent associations for every measurement, providing additional substantiation for the reliability of the outcome assessments in the present study. Third, information on dietary intake was only available among a subset of the study population, lowering the statistical power to detect potential interactions. Although dietary habits have been reported to be stable for years [48] and a single food frequency questionnaire has been used to assess dietary omega-3 and omga-6 levels in successive years [49], we cannot confirm the stability of the omega-6 to omega-3 ratios in the present population, which might result in a potential misclassification bias. Fourth, although previous damage or trauma in the upper limb could affect the present results, this was not considered because of a lack of information.

## Conclusions

To our knowledge, this is the first epidemiologic study to explore the association between phthalate biomarkers and handgrip strength. We found an inverse association between urinary phthalate metabolite concentrations and handgrip strength in the elderly. The association was stronger among individuals with a high dietary omega-6 to omega-3 ratio. Because lower muscle strength in the elderly could lead to a higher risk of falls and fracture, a critical factor with respect to longevity and well-being, the present results may have substantial public health implications in the elderly population.

## Additional file

Additional file 1: Phthalate exposure and lower handgrip strength in an elderly population: A repeated-measures study. **Table S1**. Correlations between handgrip strengths (kg) in the Korean Elderly Environmental Panel II Study (2012–2015). **Table S2**. Distribution of urinary phthalate metabolites (μg/L) in the Korean Elderly Environmental Panel II Study (2012–2015). **Table S3**. Correlations between Phthalate Metabolite Levels (μg/L) Measured at Three Surveys in the Korean Elderly Environmental Panel II Study (2012–2015). **Table S4**. Correlations between Levels of Each Phthalate Metabolites (μg/L) in the Korean Elderly Environmental Panel II Study (2012–2015). **Table S5**. Associations between Urinary Phthalate Metabolite Concentrations (Log-transformed, μg/g Creatinine) and Handgrip Strength in Multiple Pollutant Models (*n* = 892) in the Korean Elderly Environmental Panel II Study (2012–2015). **Table S6**. Associations between urinary phthalate metabolite concentrations (log transformed, μg/g creatinine) and handgrip strength, stratified by the median of the omega-6 to omega-3 ratio (*n* = 391) in the Korean Elderly Environmental Panel II Study (2012–2015). **Table S7**. Associations between urinary phthalate metabolite concentrations (log transformed, μg/g creatinine) and handgrip strength, stratified by the 90th quantile of the omega-6 to omega-3 ratio (*n* = 391) in the Korean Elderly Environmental Panel II Study (2012–2015).**Table S8**. Associations between Urinary Phthalate Metabolite Concentrations (Log-transformed, μg/g Creatinine) and Handgrip Strength after Excluding Those Who had been Diagnosed with Stroke (*n* = 1,181), Osteoporosis, or Osteoarthritis (*n* = 815). **Table S9**. Associations between urinary phthalate metabolite concentrations (log transformed, μg/g creatinine) and handgrip strength, after inverse probability weighting for follow-up (*n* = 1,228) in the Korean Elderly Environmental Panel II Study (2012–2015). **Table S10**. Associations between urinary phthalate metabolite concentrations (log transformed, μg/g creatinine) and handgrip strength stratified by the 75th quantile of the omega-6 to omega-3 ratio, after inverse probability weighting for follow-up (*n* = 391) in the Korean Elderly Environmental Panel II Study (2012–2015). (DOCX 41 kb)

## Abbreviations

BMI: Body mass index
CRP: C-reactive protein
LOD: Limit of detection
MEHHP: Mono-(2-ethyl-5-hydroxyhexyl) phthalate
MEOHP: Mono-(2-ethyl-5-oxohexyl) phthalate
MnBP: Mono-n-butyl phthalate
PPAR-γ: Peroxisome proliferator-activated receptor-γ

## References

[1] Janjua NR, Frederiksen H, Skakkebaek NE, Wulf HC, Andersson A-M (2008). Urinary excretion of phthalates and paraben after repeated whole-body topical application in humans. Int. J. Androl..

[2] Silva MJ, Barr DB, Reidy JA, Malek NA, Hodge CC, Caudill SP (2004). Urinary levels of seven phthalate metabolites in the U.S. population from the National Health and Nutrition Examination Survey (NHANES) 1999-2000. Environ. Health Perspect.

[3] Guo Y, Alomirah H, Cho H-S, Minh TB, Mohd MA, Nakata H (2011). Occurrence of phthalate metabolites in human urine from several Asian countries. Environ Sci Technol.

[4] Kim M, Song NR, Choi J-H, Lee J, Pyo H (2014). Simultaneous analysis of urinary phthalate metabolites of residents in Korea using isotope dilution gas chromatography-mass spectrometry. Sci Total Environ.

[5] Ferguson KK, Loch-Caruso R, Meeker JD (2011). Urinary phthalate metabolites in relation to biomarkers of inflammation and oxidative stress: NHANES 1999-2006. Environ Res.

[6] Hong Y-C, Park E-Y, Park M-S, Ko JA, Oh S-Y, Kim H (2009). Community level exposure to chemicals and oxidative stress in adult population. Toxicol Lett.

[7] Kim JH, Park HY, Bae S, Lim Y-H, Hong Y-C (2013). Diethylhexyl phthalates is associated with insulin resistance via oxidative stress in the elderly: a panel study. PloS One.

[8] Meeker JD, Hu H, Cantonwine DE, Lamadrid-Figueroa H, Calafat AM, Ettinger AS (2009). Urinary phthalate metabolites in relation to preterm birth in Mexico City. Environ Health Perspect.

[9] McKenzie D, Bua E, McKiernan S, Cao Z, Aiken JM, Jonathan W (2002). Mitochondrial DNA deletion mutations: a causal role in sarcopenia. Eur J Biochem. FEBS.

[10] Schaap LA, Pluijm SMF, Deeg DJH, Visser M (2006). Inflammatory markers and loss of muscle mass (sarcopenia) and strength. Am J Med.

[11] Norman K, Stobäus N, Kulka K, Schulzke J (2014). Effect of inflammation on handgrip strength in the non-critically ill is independent from age, gender and body composition. Eur J Clin Nutr.

[12] Andriollo-Sanchez M, Hininger-Favier I, Meunier N, Venneria E, O’Connor JM, Maiani G (2005). Age-related oxidative stress and antioxidant parameters in middle-aged and older European subjects: the ZENITH study. Eur J Clin Nutr.

[13] Rantanen T, Guralnik JM, Sakari-Rantala R, Leveille S, Simonsick EM, Ling S (1999). Disability, physical activity, and muscle strength in older women: the Women’s Health and Aging Study. Arch Phys Med Rehabil.

[14] Cheung C-L, Tan KCB, Bow CH, Soong CSS, Loong CHN, Kung AW-C (2012). Low handgrip strength is a predictor of osteoporotic fractures: cross-sectional and prospective evidence from the Hong Kong Osteoporosis Study. Age Dordr Neth.

[15] Cruz-Jentoft AJ, Baeyens JP, Bauer JM, Boirie Y, Cederholm T, Landi F (2010). Sarcopenia: European consensus on definition and diagnosis: Report of the European Working Group on Sarcopenia in Older People. Age Ageing.

[16] Lauretani F, Russo CR, Bandinelli S, Bartali B, Cavazzini C, Di Iorio A (2003). Age-associated changes in skeletal muscles and their effect on mobility: an operational diagnosis of sarcopenia. J Appl Physiol Bethesda Md 1985.

[17] Buser MC, Murray HE, Scinicariello F (2014). Age and sex differences in childhood and adulthood obesity association with phthalates: analyses of NHANES 2007-2010. Int J Hyg Environ Health.

[18] Hicks GE, Shardell M, Alley DE, Miller RR, Bandinelli S, Guralnik J (2012). Absolute strength and loss of strength as predictors of mobility decline in older adults: the InCHIANTI study. J Gerontol A Biol Sci Med Sci.

[19] Hirsch CH, Buzková P, Robbins JA, Patel KV, Newman AB (2012). Predicting late-life disability and death by the rate of decline in physical performance measures. Age Ageing.

[20] Gallucci M, Mazzuco S, Ongaro F, Di Giorgi E, Mecocci P, Cesari M (2013). Body mass index, lifestyles, physical performance and cognitive decline: the “Treviso Longeva (TRELONG)” study. J Nutr Health Aging.

[21] Liu H-Q, Qiu Y, Mu Y, Zhang X-J, Liu L, Hou X-H (2013). A high ratio of dietary n-3/n-6 polyunsaturated fatty acids improves obesity-linked inflammation and insulin resistance through suppressing activation of TLR4 in SD rats. Nutr Res N Y N.

[22] Strain JJ, Yeates AJ, van Wijngaarden E, Thurston SW, Mulhern MS, McSorley EM (2015). Prenatal exposure to methyl mercury from fish consumption and polyunsaturated fatty acids: associations with child development at 20 mo of age in an observational study in the Republic of Seychelles. Am J Clin Nutr.

[23] Hornung RW, Reed LD (1990). Estimation of average concentration in the presence of nondetectable values. Appl Occup Environ Hyg.

[24] Dourado VZ, de O Antunes LC, Tanni SE, De Paiva SAR, Padovani CR, Godoy I (2006). Relationship of upper-limb and thoracic muscle strength to 6-min walk distance in COPD patients. Chest..

[25] Bornehag C-G, Carlstedt F, Jönsson BAG, Lindh CH, Jensen TK, Bodin A (2015). Prenatal phthalate exposures and anogenital distance in Swedish boys. Environ Health Perspect.

[26] Meeker JD, Ferguson KK (2014). Urinary phthalate metabolites are associated with decreased serum testosterone in men, women, and children from NHANES 2011-2012. J Clin Endocrinol Metab.

[27] Sun Z, Tao Y, Li S, Ferguson KK, Meeker JD, Park SK (2013). Statistical strategies for constructing health risk models with multiple pollutants and their interactions: possible choices and comparisons. Environ Health Glob Access Sci Source.

[28] Ambring A, Johansson M, Axelsen M, Gan L, Strandvik B, Friberg P (2006). Mediterranean-inspired diet lowers the ratio of serum phospholipid n-6 to n-3 fatty acids, the number of leukocytes and platelets, and vascular endothelial growth factor in healthy subjects. Am J Clin Nutr.

[29] Broughton KS, Johnson CS, Pace BK, Liebman M, Kleppinger KM (1997). Reduced asthma symptoms with n-3 fatty acid ingestion are related to 5-series leukotriene production. Am J Clin Nutr.

[30] Robins JM, Rotnitzky A, Zhao LP (1995). Analysis of semiparametric regression models for repeated outcomes in the presence of missing data. J Am Stat Assoc.

[31] Simopoulos AP (2008). The importance of the omega-6/omega-3 fatty acid ratio in cardiovascular disease and other chronic diseases. Exp Biol Med Maywood NJ.

[32] Hurst CH, Waxman DJ (2003). Activation of PPARalpha and PPARgamma by environmental phthalate monoesters. Toxicol Sci Off J Soc Toxicol.

[33] Hotamisligil GS (2006). Inflammation and metabolic disorders. Nature.

[34] Romanyukha AA, Rudnev SG, Sidorov IA (2006). Energy cost of infection burden: an approach to understanding the dynamics of host-pathogen interactions. J Theor Biol.

[35] Chow LS, Albright RC, Bigelow ML, Toffolo G, Cobelli C, Nair KS (2006). Mechanism of insulin’s anabolic effect on muscle: measurements of muscle protein synthesis and breakdown using aminoacyl-tRNA and other surrogate measures. Am J Physiol Endocrinol Metab.

[36] Hoppin JA, Brock JW, Davis BJ, Baird DD (2002). Reproducibility of urinary phthalate metabolites in first morning urine samples. Environ Health Perspect.

[37] Hauser R, Meeker JD, Park S, Silva MJ, Calafat AM (2004). Temporal variability of urinary phthalate metabolite levels in men of reproductive age. Environ Health Perspect.

[38] Trasande L, Spanier AJ, Sathyanarayana S, Attina TM, Blustein J (2013). Urinary phthalates and increased insulin resistance in adolescents. Pediatrics.

[39] Schettler T (2006). Human exposure to phthalates via consumer products. Int J Androl.

[40] Ooi EMM, Ng TWK, Watts GF, Barrett PHR (2013). Dietary fatty acids and lipoprotein metabolism: new insights and updates. Curr. Opin. Lipidol..

[41] Schmitz G, Ecker J (2008). The opposing effects of n-3 and n-6 fatty acids. Prog Lipid Res.

[42] Frost RA, Lang CH (2011). mTor signaling in skeletal muscle during sepsis and inflammation: where does it all go wrong?. Physiol Bethesda Md.

[43] Reid WD, Rurak J, Harris RL (2009). Skeletal muscle response to inflammation--lessons for chronic obstructive pulmonary disease. Crit Care Med.

[44] Haegens A, Schols AM, Gorissen SH, van Essen AL, Snepvangers F, Gray DA (2012). NF-kB activation and polyubiquitin conjugation are required for pulmonary inflammation-induced diaphragm atrophy. Am J Physiol Lung Cell Mol Physiol.

[45] Wang C-Y, Yeh C-J, Hu M-H (2011). Mobility-related performance tests to predict mobility disability at 2-year follow-up in community-dwelling older adults. Arch Gerontol Geriatr.

[46] Sallinen J, Stenholm S, Rantanen T, Heliövaara M, Sainio P, Koskinen S (2010). Hand-grip strength cut points to screen older persons at risk for mobility limitation. J Am Geriatr Soc.

[47] Taekema DG, Gussekloo J, Maier AB, Westendorp RGJ, de Craen AJM (2010). Handgrip strength as a predictor of functional, psychological and social health. A prospective population-based study among the oldest old. Age Ageing.

[48] Goldbohm RA, van’t Veer P, van den Brandt PA, van’t Hof MA, Brants HA, Sturmans F (1995). Reproducibility of a food frequency questionnaire and stability of dietary habits determined from five annually repeated measurements. Eur J Clin Nutr.

[49] Song M, Chan AT, Fuchs CS, Ogino S, Hu FB, Mozaffarian D (2014). Dietary intake of fish, ω-3 and ω-6 fatty acids and risk of colorectal cancer: A prospective study in U.S. men and women. Int J Cancer.

