# Supplementary material for: Association between phthalate exposure and lower handgrip strength in an elderly population: a repeated-measures study
Source: Environ Health. 2016 Aug 31;15(1):93. doi: 10.1186/s12940-016-0176-2 (PMC5006265; doi:10.1186/s12940-016-0176-2)
Supplement: Additional file 1: — Phthalate exposure and lower handgrip strength in an elderly population: A repeated-measures study. Table S1. Correlations between handgrip strengths (kg) in the Korean Elderly Environmental Panel II Study (2012–2015). Table S2. Distribution of urinary phthalate metabolites (μg/L) in the Korean Elderly Environmental Panel II Study (2012–2015). Table S3. Correlations between Phthalate Metabolite Levels (μg/L) Measured at Three Surveys in the Korean Elderly Environmental Panel II Study (2012–2015). Table S4. Correlations between Levels of Each Phthalate Metabolites (μg/L) in the Korean Elderly Environmental Panel II Study (2012–2015). Table S5. Associations between Urinary Phthalate Metabolite Concentrations (Log-transformed, μg/g Creatinine) and Handgrip Strength in Multiple Pollutant Models (n = 892) in the Korean Elderly Environmental Panel II Study (2012–2015). Table S6. Associations between urinary phthalate metabolite concentrations (log transformed, μg/g creatinine) and handgrip strength, stratified by the median of the omega-6 to omega-3 ratio (n = 391) in the Korean Elderly Environmental Panel II Study (2012–2015). Table S7. Associations between urinary phthalate metabolite concentrations (log transformed, μg/g creatinine) and handgrip strength, stratified by the 90th quantile of the omega-6 to omega-3 ratio (n = 391) in the Korean Elderly Environmental Panel II Study (2012–2015).Table S8. Associations between Urinary Phthalate Metabolite Concentrations (Log-transformed, μg/g Creatinine) and Handgrip Strength after Excluding Those Who had been Diagnosed with Stroke (n = 1,181), Osteoporosis, or Osteoarthritis (n = 815). Table S9. Associations between urinary phthalate metabolite concentrations (log transformed, μg/g creatinine) and handgrip strength, after inverse probability weighting for follow-up (n = 1,228) in the Korean Elderly Environmental Panel II Study (2012–2015). Table S10. Associations between urinary phthalate metabolite concentrations (log trans [file 12940_2016_176_MOESM1_ESM.docx]

**Supplementary Material: Phthalate exposure and lower handgrip strength in an elderly population: A repeated-measures study**

Kyoung-Nam Kim, Mee-Ri Lee, Yoon-Hyeong Choi, Hyojung Hwang, Se-Young Oh, ChoongHee Park, and Yun-Chul Hong

**Table of Contents:**

**Table S1.** Correlations between handgrip strengths (kg) in the Korean Elderly Environmental Panel II Study (2012–2015)

**Table S2.** Distribution of urinary phthalate metabolites (μg/L) in the Korean Elderly Environmental Panel II Study (2012–2015)

**Table S3.** Correlations between Phthalate Metabolite Levels (μg/L) Measured at Three Surveys in the Korean Elderly Environmental Panel II Study (2012–2015)

**Table S4.** Correlations between Levels of Each Phthalate Metabolites (μg/L) in the Korean Elderly Environmental Panel II Study (2012–2015)

**Table S5.** Associations between Urinary Phthalate Metabolite Concentrations (Log-transformed, μg/g Creatinine) and Handgrip Strength in Multiple Pollutant Models (*n* = 892) in the Korean Elderly Environmental Panel II Study (2012–2015)

**Table S6.** Associations between urinary phthalate metabolite concentrations (log transformed, μg/g creatinine) and handgrip strength, stratified by the median of the omega-6 to omega-3 ratio (*n* = 391) in the Korean Elderly Environmental Panel II Study (2012–2015)

**Table S7.** Associations between urinary phthalate metabolite concentrations (log transformed, μg/g creatinine) and handgrip strength, stratified by the 90^th^ quantile of the omega-6 to omega-3 ratio (*n* = 391) in the Korean Elderly Environmental Panel II Study (2012–2015)

**Table S8.** Associations between Urinary Phthalate Metabolite Concentrations (Log-transformed, μg/g Creatinine) and Handgrip Strength after Excluding Those Who had been Diagnosed with Stroke (*n* = 1,181), Osteoporosis, or Osteoarthritis (*n* = 815)

**Table S9.** Associations between urinary phthalate metabolite concentrations (log transformed, μg/g creatinine) and handgrip strength, after inverse probability weighting for follow-up (*n* = 1,228) in the Korean Elderly Environmental Panel II Study (2012–2015)

**Table S10.** Associations between urinary phthalate metabolite concentrations (log transformed, μg/g creatinine) and handgrip strength stratified by the 75^th^ quantile of the omega-6 to omega-3 ratio, after inverse probability weighting for follow-up (*n* = 391) in the Korean Elderly Environmental Panel II Study (2012–2015)

**Table S1.** Correlations between handgrip strengths (kg) in the Korean Elderly Environmental Panel II Study (2012–2015)

|  | 1^st^ attempt  with right hand | 2^nd^ attempt  with right hand | 1^st^ attempt  with left hand | 2^nd^ attempt  with left hand |
| --- | --- | --- | --- | --- |
| 1^st^ attempt  with right hand | 1.00 | 0.96  (< 0.01) | 0.92  (< 0.01) | 0.90  (< 0.01) |
| 2^nd^ attempt  with right hand |  | 1.00 | 0.92  (< 0.01) | 0.91  (< 0.01) |
| 1^st^ attempt  with left hand |  |  | 1.00 | 0.97  (< 0.01) |
| 2^nd^ attempt  with left hand |  |  |  | 1.00 |

Data are shown as Pearson’s correlation coefficients (*p*-value).

**Table S2.** Distribution of urinary phthalate metabolites (μg/L) in the Korean Elderly Environmental Panel II Study (2012–2015)

| Metabolite | LOD | <LOD (%) | GM | GSD | Percentile | | | | |
| --- | --- | --- | --- | --- | --- | --- | --- | --- | --- |
|  |  |  |  |  | 5th | 25th | 50th | 75th | 95th |
| MEOHP | 0.32 | 0.33 | 15.48 | 2.50 | 3.72 | 9.17 | 16.07 | 27.50 | 61.54 |
| MEHHP | 0.20 | 0 | 21.11 | 2.53 | 4.87 | 12.34 | 21.64 | 37.90 | 87.41 |
| MnBP | 0.35 | 0 | 28.23 | 2.17 | 7.56 | 17.00 | 28.90 | 45.73 | 93.20 |

Abbreviations: MEOHP, mono-(2-ethyl-5-oxohexyl) phthalate; MEHHP, mono-(2-ethyl-5-hydroxyhexyl) phthalate; MnBP, mono-n-butyl-phthalate; LOD, limit of detection; GM, geometric mean; GSD, geometric standard deviation.

**Table S3.** Correlations between Phthalate Metabolite Levels (μg/L) Measured at Three Surveys in the Korean Elderly Environmental Panel II Study (2012–2015)

|  | First survey | Second survey | Third survey |
| --- | --- | --- | --- |
| MEOHP |  |  |  |
| First survey | 1.00 | 0.44 (< 0.01) | 0.44 (< 0.01) |
| Second survey |  | 1.00 | 0.50 (< 0.01) |
| Third survey |  |  | 1.00 |
| MEHHP |  |  |  |
| First survey | 1.00 | 0.45 (< 0.01) | 0.44 (< 0.01) |
| Second survey |  | 1.00 | 0.54 (< 0.01) |
| Third survey |  |  | 1.00 |
| MnBP |  |  |  |
| First survey | 1.00 | 0.20 (< 0.01) | 0.30 (< 0.01) |
| Second survey |  | 1.00 | 0.28 (< 0.01) |
| Third survey |  |  | 1.00 |

Abbreviations: MEOHP, mono-(2-ethyl-5-oxohexyl) phthalate; MEHHP, mono-(2-ethyl-5-hydroxyhexyl) phthalate; MnBP, mono-n-butyl-phthalate.

Data are shown as Pearson’s correlation coefficients (*p*-value).

**Table S4.** Correlations between Levels of Each Phthalate Metabolites (μg/L) in the Korean Elderly Environmental Panel II Study (2012–2015)

|  | MEOHP | MEHHP | MnBP |
| --- | --- | --- | --- |
| First survey |  |  |  |
| MEOHP | 1.00 | 0.94 (<0.01) | 0.48 (<0.01) |
| MEHHP |  | 1.00 | 0.44 (<0.01) |
| MnBP |  |  | 1.00 |
| Second survey |  |  |  |
| MEOHP | 1.00 | 0.90 (<0.01) | 0.28 (<0.01) |
| MEHHP |  | 1.00 | 0.26 (<0.01) |
| MnBP |  |  | 1.00 |
| Third survey |  |  |  |
| MEOHP | 1.00 | 0.92 (<0.01) | 0.36 (<0.01) |
| MEHHP |  | 1.00 | 0.36 (<0.01) |
| MnBP |  |  | 1.00 |

Abbreviations: MEOHP, mono-(2-ethyl-5-oxohexyl) phthalate; MEHHP, mono-(2-ethyl-5-hydroxyhexyl) phthalate; MnBP, mono-n-butyl-phthalate.

Data are shown as Pearson’s correlation coefficients (*p*-value).

**Table S5.** Associations between Urinary Phthalate Metabolite Concentrations (Log-transformed, μg/g Creatinine) and Handgrip Strength in Multiple Pollutant Models^a^ (*n* = 892)^b^ in the Korean Elderly Environmental Panel II Study (2012–2015)

|  | ∑DEHP | |  | MnBP | |
| --- | --- | --- | --- | --- | --- |
|  | β | 95% CI |  | β | 95% CI |
| Right hand | –0.21 | –0.32, –0.09 |  | –0.27 | –0.38, –0.15 |
| Left hand | –0.26 | –0.38, –0.14 |  | –0.18 | –0.29, –0.08 |

Abbreviations: ∑DEHP , the molar sum of mono-(2-ethyl-5-oxohexyl) phthalate and mono-(2-ethyl-5-hydroxyhexyl) phthalate; MnBP, mono-n-butyl-phthalate; CI, confidence interval.

^a^Least absolute shrinkage and selection operator (LASSO) regression models that included age, sex, smoking status, alcohol consumption, physical activity, monthly income, education level, city of residence, body mass index, comorbidity status, blood lead concentration, blood mercury concentration, blood cadmium concentration, log-transformed creatinine-adjusted urinary 3-phenoxybenzoic acid concentration, ∑DEHP, and MnBP. ^b^The number of subjects was less than that of the main analysis because of missing environmental pollutant level data.

**Table S6.** Associations^a^ between urinary phthalate metabolite concentrations (log transformed, μg/g creatinine) and handgrip strength, stratified by the median of the omega-6 to omega-3 ratio (*n* = 391) in the Korean Elderly Environmental Panel II Study (2012–2015)

|  | MEOHP | | |  | MEHHP | | |  | MnBP | | |
| --- | --- | --- | --- | --- | --- | --- | --- | --- | --- | --- | --- |
| Ratio^b^ | β | 95% CI | *p*-int |  | β | 95% CI | *p*-int |  | β | 95% CI | *p*-int |
|  | Right hand | | | | | | | | | | |
| High | –1.03 | –1.62, –0.44 | 0.5157 |  | –1.03 | –1.66, –0.41 | 0.3813 |  | –1.09 | –1.76, –0.42 | 0.2399 |
| Low | –0.79 | –1.35, –0.22 |  |  | –0.68 | –1.30, –0.05 |  |  | –0.32 | –1.05, 0.40 |  |
|  | Left hand | | | | | | | | | | |
| High | –0.74 | –1.35, –0.14 | 0.9754 |  | –0.72 | –1.36, –0.08 | 0.8872 |  | –0.93 | –1.62, –0.25 | 0.4768 |
| Low | –0.79 | –1.33, –0.25 |  |  | –0.68 | –1.28, –0.09 |  |  | –0.37 | –1.06, 0.32 |  |

Abbreviations: MEOHP, mono-(2-ethyl-5-oxohexyl) phthalate; MEHHP, mono-(2-ethyl-5-hydroxyhexyl) phthalate; MnBP, mono-n-butyl-phthalate; CI, confidence interval; *p*-int, *p*-value for interaction.

^a^Adjusted for age, sex, smoking status, alcohol consumption, physical activity, monthly income, education level, city of residence, body mass index, and comorbidity status. ^b^Omega-6 to omega-3 ratios above and below the median (7.54) are defined as high and low, respectively.

**Table S7.** Associations^a^ between urinary phthalate metabolite concentrations (log transformed, μg/g creatinine) and handgrip strength, stratified by the 90^th^ quantile of the omega-6 to omega-3 ratio (*n* = 391) in the Korean Elderly Environmental Panel II Study (2012–2015)

|  | MEOHP | | |  | MEHHP | | |  | MnBP | | |
| --- | --- | --- | --- | --- | --- | --- | --- | --- | --- | --- | --- |
| Ratio^b^ | β | 95% CI | *p*-int |  | β | 95% CI | *p*-int |  | β | 95% CI | *p*-int |
|  | Right hand | | | | | | | | | | |
| High | –2.24 | –3.71, –0.78 | 0.0930 |  | –1.95 | –3.61, –0.29 | 0.1564 |  | –1.12 | –2.40, 0.16 | 0.3876 |
| Low | –0.83 | –1.26, –0.40 |  |  | –0.84 | –1.30, –0.38 |  |  | –0.79 | –1.33, –0.25 |  |
|  | Left hand | | | | | | | | | | |
| High | –2.11 | –3.74, –0.48 | 0.0697 |  | –2.01 | –3.82, –0.21 | 0.0786 |  | –0.81 | –2.22, 0.60 | 0.4297 |
| Low | –0.67 | –1.09, –0.25 |  |  | –0.64 | –1.09, –0.19 |  |  | –0.71 | –1.24, –0.18 |  |

Abbreviations: MEOHP, mono-(2-ethyl-5-oxohexyl) phthalate; MEHHP, mono-(2-ethyl-5-hydroxyhexyl) phthalate; MnBP, mono-n-butyl-phthalate; CI, confidence interval; *p*-int, *p*-value for interaction.

^a^Adjusted for age, sex, smoking status, alcohol consumption, physical activity, monthly income, education level, city of residence, body mass index, and comorbidity status. ^b^Omega-6 to omega-3 ratios above and below the 90^th^ quantile value (9.94) are defined as high and low, respectively.

**Table S8.** Associations^a^ between Urinary Phthalate Metabolite Concentrations (Log-transformed, μg/g Creatinine) and Handgrip Strength after Excluding Those Who had been Diagnosed with Stroke (*n* = 1,181), Osteoporosis, or Osteoarthritis (*n* = 815)

|  | MEOHP | |  | MEHHP | |  | MnBP | |
| --- | --- | --- | --- | --- | --- | --- | --- | --- |
|  | β | 95% CI |  | β | 95% CI |  | β | 95% CI |
| Those without previous stroke (*n* = 1,181) | | | | | | | | |
| Right hand^b^ | −0.66 | −0.96, −0.37 |  | −0.58 | −0.89, −0.28 |  | −0.52 | −0.87, −0.17 |
| Left hand^b^ | −0.62 | −0.91, −0.33 |  | −0.51 | −0.81, −0.21 |  | −0.48 | −0.82, −0.14 |
| Those without previous stroke, osteoporosis, or osteoarthritis (*n* = 815) | | | | | | | | |
| Right hand^b^ | −0.66 | −1.04, −0.29 |  | −0.49 | −0.88, −0.10 |  | −0.51 | −0.99, −0.04 |
| Left hand^b^ | −0.65 | −1.02, −0.27 |  | −0.49 | −0.88, −0.11 |  | −0.57 | −1.04, −0.10 |

MEOHP, mono-(2-ethyl-5-oxohexyl) phthalate; MEHHP, mono-(2-ethyl-5-hydroxyhexyl) phthalate; MnBP, mono-n-butyl-phthalate; CI, confidence interval.

^a^Adjusted for age, sex, smoking status, alcohol consumption, physical activity, monthly income, education level, city of residence, body mass index, and comorbidity status. ^b^Average of the handgrip strengths measured at the first and second attempt.

**Table S9.** Associations^a^ between urinary phthalate metabolite concentrations (log transformed, μg/g creatinine) and handgrip strength, after inverse probability weighting for follow-up (*n* = 1,228) in the Korean Elderly Environmental Panel II Study (2012–2015)

|  | MEOHP | |  | MEHHP | |  | MnBP | |
| --- | --- | --- | --- | --- | --- | --- | --- | --- |
|  | β | 95% CI |  | β | 95% CI |  | β | 95% CI |
| Right hand | –0.63 | –0.92, –0.35 |  | –0.63 | –0.93, –0.33 |  | –0.47 | –0.80, –0.13 |
| Right hand 1 | –0.66 | –0.95, –0.36 |  | –0.65 | –0.95, –0.34 |  | –0.53 | –0.88, –0.19 |
| Right hand 2 | –0.61 | –0.90, –0.32 |  | –0.61 | –0.92, –0.30 |  | –0.40 | –0.75, –0.05 |
| Left hand | –0.52 | –0.81, –0.24 |  | –0.51 | –0.81, –0.21 |  | –0.51 | –0.84, –0.17 |
| Left hand 1 | –0.56 | –0.85, –0.26 |  | –0.55 | –0.86, –0.24 |  | –0.53 | –0.87, –0.18 |
| Left hand 2 | –0.49 | –0.78, –0.20 |  | –0.48 | –0.78, –0.17 |  | –0.48 | –0.83, –0.14 |

Abbreviations: MEOHP, mono-(2-ethyl-5-oxohexyl) phthalate; MEHHP, mono-(2-ethyl-5-hydroxyhexyl) phthalate; MnBP, mono-n-butyl-phthalate; CI, confidence interval.

^a^Adjusted for age, sex, smoking status, alcohol consumption, physical activity, monthly income, education level, city of residence, body mass index, and comorbidity status.

**Table S10.** Associations^a^ between urinary phthalate metabolite concentrations (log transformed, μg/g creatinine) and handgrip strength stratified by the 75^th^ quantile of the omega-6 to omega-3 ratio, after inverse probability weighting for follow-up (*n* = 391) in the Korean Elderly Environmental Panel II Study (2012–2015)

|  | MEOHP | | |  | MEHHP | | |  | MnBP | | |
| --- | --- | --- | --- | --- | --- | --- | --- | --- | --- | --- | --- |
| Ratio^b^ | β | 95% CI | *p*-int |  | β | 95% CI | *p*-int |  | β | 95% CI | *p*-int |
|  | Right hand | | | | | | | | | | |
| High | –1.73 | –2.62, –0.85 | 0.0457 |  | –1.53 | –2.53, –0.53 | 0.1430 |  | –1.46 | –2.40, –0.53 | 0.1216 |
| Low | –0.62 | –1.10, –0.14 |  |  | –0.70 | –1.22, –0.18 |  |  | –0.60 | –1.19, –0.01 |  |
|  | Left hand | | | | | | | | | | |
| High | –1.25 | –2.15, –0.35 | 0.2022 |  | –1.16 | –2.17, –0.15 | 0.3396 |  | –1.35 | –2.30, –0.40 | 0.1272 |
| Low | –0.55 | –1.02, –0.09 |  |  | –0.61 | –1.11, –0.10 |  |  | –0.57 | –1.14, 0.01 |  |

Abbreviations: MEOHP, mono-(2-ethyl-5-oxohexyl) phthalate; MEHHP, mono-(2-ethyl-5-hydroxyhexyl) phthalate; MnBP, mono-n-butyl-phthalate; CI, confidence interval; *p*-int, *p*-value for interaction.

^a^Adjusted for age, sex, smoking status, alcohol consumption, physical activity, monthly income, education level, city of residence, body mass index, and comorbidity status. ^b^Omega-6 to omega-3 ratios above and below the 75^th^ quantile value (8.81) are defined as high and low, respectively.
